# Brownfields to Greenfields: Environmental Justice Versus Environmental Gentrification

**DOI:** 10.3390/ijerph15102233

**Published:** 2018-10-12

**Authors:** Juliana A. Maantay, Andrew R. Maroko

**Affiliations:** 1 Department of Earth, Environmental, and Geospatial Sciences, Lehman College, City University of New York, New York, NY 10468, USA; 2 CUNY Graduate School of Public Health and Health Policy, City University of New York, New York, NY 10027, USA; Andrew.maroko@sph.cuny.edu; 3 The CUNY Graduate Center, Earth and Environmental Science Doctoral Program, City University of New York, New York, NY 10016, USA

**Keywords:** environmental justice, community gardens, spatial analysis, gentrification, Brooklyn, Vacant Land, GIS

## Abstract

Gentrification is a growing concern in many urban areas, due to the potential for displacement of lower-income and other vulnerable populations. This process can be accelerated when neighborhood “greening” projects are undertaken via governmental or private investor efforts, resulting in a phenomenon termed environmental or “green” gentrification. Vacant land in lower-income areas is often improved by the existing community through the creation of community gardens, but this contributes to these greening efforts and paradoxically may spur gentrification and subsequent displacement of the gardens’ stewards and neighbors. “Is proximity to community gardens in less affluent neighborhoods associated with an increased likelihood of gentrification?” Using Brooklyn, New York as a case study, we examined this question using Geographic Information Systems and two spatial methods: a census block group proximity analysis, and a hot spot analysis, to determine the potential impact of proximity to community gardens in lower-income areas. The results of the analyses suggest that proximity to community gardens is associated with significant increases in per capita income over the five years study period, which is indicative of areas undergoing gentrification. This has implications for environmental justice because existing lower-income residents are likely to be displaced after their community is improved environmentally.

## 1. Introduction

Many post-industrial cities have high proportions of vacant and derelict land (VDL), which have numerous deleterious environmental and health impacts on the proximate populations [1,2,3]. VDL can be put to beneficial use for communities, such as urban agriculture, passive or active recreation spaces, farmers’ markets, natural areas connecting with existing open space networks, or urban forestry phyto-remediation. Typically, VDL is located predominantly in poorer neighborhoods, presenting a disproportionate environmental and health risk to these more vulnerable populations—risks that could be mitigated or reduced by constructive re-use. Conversely, re-use of VDL also poses risks to these communities, due to the potential for displacing poor people and marginalized groups through gentrification, which often follows community greening efforts and also may stimulate developer- or government-initiated green infrastructure projects and other large developments, potentially detrimental to the existing community.

In less affluent parts of many urban areas, there is typically more vacant and derelict property than in middle-class or higher income neighborhoods. These vacant lots represent where perhaps industry used to be located, but which has now relocated elsewhere or become obsolete, such as in Glasgow [3], or where widespread housing abandonment by absentee landlords took place, such as in parts of New York City (NYC) [4]. Generally speaking, more affluent areas do not have land remaining vacant for long in their neighborhoods, because land there is too valuable to leave fallow. In poorer areas or within communities of color, vacant land is more common, and may stay unused for decades due to the lack of private developer interest and/or the absence of governmental will for public investment in these areas. Due to the high cost of re-developing vacant land, especially land that has been contaminated by prior industrial uses, such brownfield redevelopment is normally undertaken where there is a strong likelihood of making a profit, or in the case of governmental interventions, of increasing the tax rolls. Affordable housing and other uses for the existing community’s benefit usually do not fall into this high profit-making category and does not therefore justify the risk and expense involved, so vacant land can remain vacant for a long time in less affluent areas. However, once the process of gentrification begins in a neighborhood, or there is a spill-over gentrification effect from an adjacent or nearby neighborhood, vacant lots can become attractive to developers for higher-end residential or even commercial construction, all of which often involves providing green amenities as part of the project to attract the desired affluent consumer market to the area.

“Brownfield redevelopment projects often emphasise economic development and may produce neighbourhood-wide increases in property values, decreased availability of affordable housing, and changes in commercial/retail presence. Such changes can contribute to quality of life improvements and reduce place-based vulnerabilities associated with soil contamination; however, increases in the cost of living associated with remediation and redevelopment have the potential to facilitate gentrification” [5] (p. 1017).

Although the “gentrification” phenomenon has been much remarked upon and studied for at least the past 50 years, and the process itself is far older, it is still a difficult term to define, and even more difficult to measure. Analyses and case studies of the gentrification process discuss various definitions of gentrification but there is no general consensus of how to definitively identify communities undergoing gentrification [6,7,8]. For just two such examples of researchers’ attempts to pin down the definition, Atkinson defined gentrification as “a process of class succession and displacement in areas broadly characterized by working-class and unskilled households” [9] (p. 149). Clark defined it as a process resulting in “increased proportions of residents of a higher socio-economic status in concert with reinvestment of the built environment” [10] (p. 258).

Gentrification can be thought of as referring to a significant change (referred to by Hwang as “upgrading”) in an area’s socioeconomic characteristics, a change in the physical environment (housing stock, infrastructure, and amenities), an overall change in neighborhood culture and economics, and any combination of the above, any of which is likely to inexorably lead to displacement of the original neighborhood residents and businesses and their replacement by more affluent residents and up-scale businesses [11] (p. 1). In most cases in the United States, the existing/displaced residents are people of color, immigrants, ethnic minorities, or lower-income and working-class Whites, and the residents who replace them are usually more affluent non-Hispanic Whites.

Very often, the data used to measure gentrification inform how gentrification is defined, with analyses using more quantitative data, especially census data, focusing more on the sociodemographic and economic changes [12], and approaches using mainly qualitative data tend to define gentrification in terms of cultural changes and changes in housing stock and other visible aspects of the built environment [5,13]. The quantitative approaches usually look at changes over time in household income, educational levels, rent or home prices, percentage of non-Hispanic White population, unemployment rates, and sometimes percentage of the adult population employed in professional jobs. These are analyzed as individual variables, or used in some combination to create an index. Often, studies categorize certain census tracts or neighborhoods as being “eligible” for gentrification—meaning that the tracts are not already middle-class or higher—to distinguish neighborhoods which may have experienced significant change during the study time period, but are not in danger of gentrification, or have already been substantially gentrified [11].

Gentrification is a phenomenon of concern because in most urban areas today (especially cities in the US and other western industrial or post-industrial countries) housing opportunities in working class and lower-income neighborhoods are already scarce, and overcrowding is common. This is exacerbated in cities having large racial or ethnic minority populations, due to the historic consequences of racial/ethnic segregation imposed by custom, public policy, and law, as well as by the more limited financial options of the minority populations [14,15]. Eventually in areas where gentrification begins, and sometimes sooner rather than later, rents are raised, housing prices increase, and subsequently existing residents are forced out. Gentrification has adverse implications for small businesses in the neighborhood, as well, and often within a relatively short time the changes in residents and businesses alters the entire cultural landscape of the community, to the point that, even if the original residents are financially able to stay, they frequently do not feel like they belong in their neighborhood anymore [5,14].

Since the 1960s in New York City and elsewhere, and increasing throughout the 1970s–1990s, community-based grassroots groups have been turning to the vacant and derelict land in their neighborhoods to create additional green space and bring other advantages to their communities, such as VDL lots converted to green spaces serving as a locus for youth and environmental programs, cultural events, space for the performing arts, healthy food production, inter-generational activities, inter-racial cooperation, knowledge transfer, and a means of political and social empowerment and engagement for the community [4,16,17,18,19,20]. Approximately 5% of New York City’s nearly 30,000 vacant property lots (equaling about 7000 acres) are used as community gardens, and most of those are on publicly-owned land [21].

It has been long-recognized that urban green spaces are important to individuals’ physical and mental health, as well as for community well-being, cohesion, and resilience [22,23,24,25,26,27]. A comprehensive summary of publications studying the relationship between health and green space is provided in Table 1 of the Jennings et al. (2016) paper in this volume [28] (p. 3). Some research has also shown that poorer and more minority neighborhoods often have less access to open space, and that the open space they do have tends to be smaller or of an inferior quality than that in more affluent neighborhoods [29,30,31,32,33], although not all studies support these conclusions [34,35,36]. Additionally, in more affluent areas, people are more likely to have personal vehicles which can be used to access distant open space such as state and national parks and forests, an option not available to many within the poorer neighborhoods. Ironically, this makes the open spaces in poorer neighborhoods even more important to the residents since they have fewer options outside their neighborhoods, and in densely-settled areas with multi-family dwellings, may be less likely to have private backyards. Thus, creating new green spaces within poorer communities would naturally be considered a good thing in providing a healthy amenity to an under-served population, and furthering environmental justice goals, which includes the concept that everyone has the right to live and work in safe and healthy environments, without disproportionate exposure to environmental hazards, and with equal access to beneficial environments [37,38].

Despite the benefits for residents in developing their own communal green spaces, the community’s conversion of derelict land, often weed- and vermin-infested, garbage-filled vacant lots, to attractive community-led green spaces, can result in unintended adverse impacts on the community, especially related to rises in property values due to new-found developer interest in the area. “A major issue in NYC with re-use of vacant and derelict land for development is the displacement of poor people through gentrification. Ironically, this has often occurred in areas where community gardens have improved property values, enhanced neighborhood aesthetics, and reduced crime rates sufficiently to interest developers in investing in the neighborhood, whereby the community rightfully feels as though their hard work has sown the seeds of their own destruction” [3] (p. 40). This is what Checker termed a “pernicious paradox—must they reject environmental amenities in their neighborhood in order to resist gentrification that tends to follow from such amenities?” [39] (p. 211). Such gentrification has been called “green” or environmental gentrification, since it stems from greening improvements.

Governmental and private developer greening projects have involved taking formerly working waterfronts and reinventing them as new destinations, spruced up with promenades, hotels, luxury residential towers, restaurants, up-scale retail stores, and open space, as well as the “park-ification” of disused rail lines and other linear infrastructure. This has created wide-spread environmental or “green” gentrification in areas adjacent to them [14,40], including greening projects such as New York City’s High Line Park and Atlanta’s BeltLine [41]. However, there have also been many anecdotal reports on the idea that community initiatives in creating low-key gardens and other small beautification improvements in less affluent neighborhoods can similarly lead to green gentrification and displacement of the existing residents and businesses [42,43,44,45]. In many cities, this seems to have been the case, although it is a complicated relationship, with a less than straightforward trajectory. In some areas of New York City, for instance, especially in the 1980s and 1990s, the gardens themselves were subjected to demolition and their lots appropriated for redevelopment for luxury housing and the like [4,17]. In more recent years, rather than developers destroying the gardens, the gardens have themselves become one of the “draws” for gentrification, with the eco-urbanism and sustainability aspirations of the prospective gentrifiers finding the existence of nearby gardens desirable. “Materially, the efforts of environmental justice activists to improve their neighborhoods (i.e., the removal of environmental burdens and the installation of environmental benefits) now help those neighborhoods attract an influx of affluent residents” [39] (p. 212).

There is an environmental justice component to environmental gentrification, in that VDL re-development can result in an actual decrease of poor and minority populations in those neighborhoods [46]. The displaced population must often relocate to worse neighborhoods—worse in terms of having more hazardous environmental conditions as well as overcrowded and less salubrious housing choices. In New York City, brownfield revitalization was found to increase stressors like geographic displacement in minority populations [47], and in Toronto, Victoria, and Vancouver, Canada, an inverse relationship was found between greening of VDL and affordability of communities [48].

Obviously, the existence of community gardens is not the only factor that spurs on gentrification. Very often it is a combination of such community-led improvement initiatives, governmental policies such as re-zoning areas to their “highest and best use”, tax incentives for adaptive re-use of derelict or abandoned building complexes, and private investment dollars looking for a likely place to land. Making the community more attractive, “greener”, does help encourage this trend, and community gardens have also been related to a reduction in crime rates [49,50,51], making the area even more feasible for outside investment. By definition, gentrification can only take place in areas that are less affluent or more working-class, often with a higher percentage of racial/ethnic minorities than the city as a whole, and preferably areas with decent transportation links to the central business district, and/or sufficient renovate-able existing housing stock or vacant land upon which to build new housing.

The need to combat this undesirable sequence of events of neighborhood greening leading to gentrification has resulted in the development of the “Just Green Enough” approach to the re-use of vacant land, “a strategy….to achieve environmental remediation without environmental gentrification” [52] (p. 1027). This strategy encourages the community-led re-use of VDL or other marginal open space for purposes that will presumably not make the area more attractive to gentrification [53], and new research has suggested simple ways to accomplish this creation of informal urban green space [54]. Community gardens could be considered a “just green enough” strategy, because they are community-led efforts, are intended for the sole purpose of benefitting the neighborhood’s people and environment, and take advantage of unutilized VDL.

To further investigate the influence of community gardens on gentrification, we used Brooklyn, New York as a case study area. We asked the question: “Is proximity to community gardens in less affluent neighborhoods associated with an increased likelihood of gentrification?”

## 2. Materials and Methods

**Figure 1:**
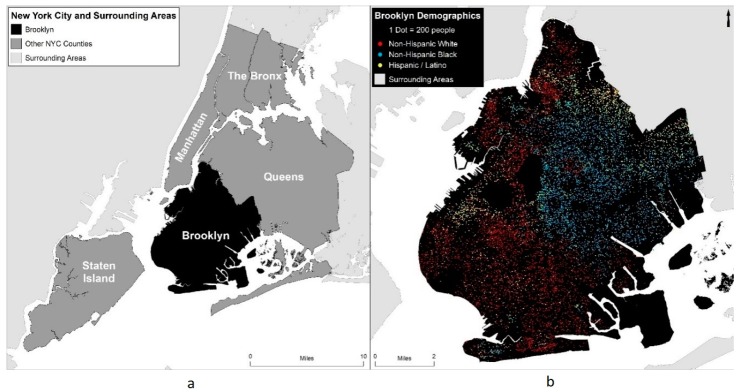
(**a**) Location of Brooklyn, NY within New York City; and(**b**) race and ethnicity distribution in Brooklyn (2010).

In this study, we compared changes in per capita income between 2010 and 2015 for census block groups in Brooklyn, NY based on their proximity to community gardens. Brooklyn, which is one of the five boroughs of New York City, was selected as our case study area due to its large geographic sections of rapid gentrification over recent years [14,55,56]. It is well connected with Manhattan via public transportation and has the second highest population density in the city [57]. Based on American Community Survey (ACS) five-year estimates, it is a demographically heterogeneous borough with respect to race and ethnicity, yet still having noteworthy residential segregation (see Figure 1). The population is comprised of 35.6% non-Hispanic White, 32.8% non-Hispanic Black, and 19.8% Hispanic/Latinx residents in 2010 and has grown by over 120,000 residents between 2010 and 2015, with a current total of 2.6 million residents in 2015 [57].

Using the framework of Geographic Information Systems (GIS), we conducted spatial analyses on several datasets. Demographic data for Brooklyn were derived from American Community Survey data via NHGIS [57]. Data included per capita income, race and ethnicity, and total population for 2006–2010 and 2011–2015 (five-year estimates) at the census block group level and borough-wide (i.e., all of Brooklyn). Per capita income from 2010 was adjusted to constant 2015 dollars using a factor of 1.08 from the Bureau of Labor Statistics calculator [58]. Change in per capita income was calculated as 2015 income minus 2010 income in (2015-adjusted dollars).

**Figure 2:**
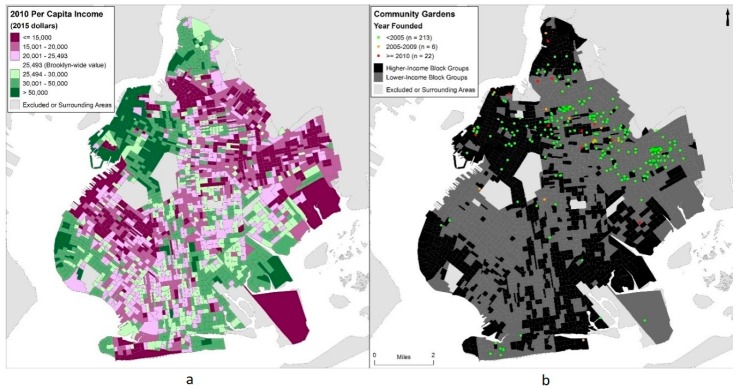
(**a**) Per capita income (2010) in Brooklyn, NY with colors diverging from the Brooklyn-wide per capita income estimate ($25,493 in 2015-adjusted dollars). (**b**) Locations of community gardens shown by year founded and lower-income and higher-income census block groups (2010).

An additional binary field was created to represent block groups which had a per capita income in 2010 below the Brooklyn-wide per capita income ($25,493 in 2015-adjusted dollars), to identify lower-income block groups which, in this study, are being considered at higher risk of gentrification [11], and as such areas that are more affluent, or have already been substantially gentrified, have been excluded (see Figure 2). Lower-income block groups were demographically distinct from the rest of Brooklyn with higher proportions of racial and ethnic minorities. For example, non-Hispanic White residents comprised over 50% of the population in higher-income block groups but only approximately 26% of residents in lower-income block groups in 2010.

Community garden locations and attributes were acquired from the Open Accessible Space Information System (OasisNYC) online mapping service. This is a comprehensive spatial data resource that has been providing data online about New York City for over 15 years. Data are compiled from information provided by governmental, non-profit, and corporate partners of OASIS and are freely available to all [59]. Brooklyn contains 241 (44%) of the 549 community gardens in the dataset. Garden locations, provided as polygons, were converted to centroids for analysis. Gardens were classified in this study as “all” (those founded in any year), “2005+” (those founded in 2005 or later), and “2010+” (those founded in 2010 or later). Gardens with missing data (*n* = 52) were included in the “all” category. This differentiation in “year founded” was made to distinguish between communities with any historical involvement with community gardens, including long-standing community engagement, from those with more recent activity (see Figure 2).

**Figure 3:**
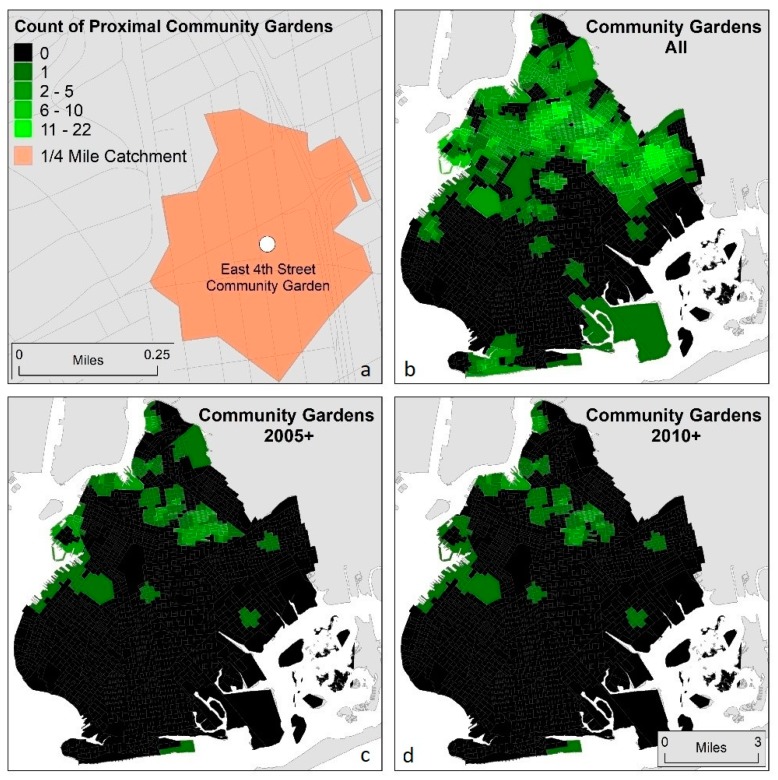
(**a**) The ¼ mile pedestrian-accessible catchment area (based on network analysis) around one community garden (East 4th Street Community Garden); (**b**) counts of number of community gardens (founded any year) within ¼ mile of each block group; (**c**) counts of number of community gardens (founded in 2005 or later) within ¼ mile of each block group; and(**d**) counts of number of community gardens (founded in 2010 or later) within ¼ mile of each block group.

Geographic proximity to environmentally burdensome or beneficial locations can be measured and conceptualized in a multitude of ways including kernel density estimation, fixed-distance buffers, and point-in-polygon analysis; however, network distances have been shown to be a reliable and widely used metric [60,61,62,63].“Network buffers”, or catchment areas, delineate areas within a specified distance (or travel time) from a location along a traversable network, such as streets. To estimate the number of community gardens proximal to block groups in Brooklyn, catchment areas were created around each garden using pedestrian accessible paths based on street files [63]. Proximity was operationalized as block groups within ¼ mile (402 m) of a community garden. The ¼ mile network distance represents a commonly-used standard for walking distance in urban areas, and as such indicates a reasonable assumption of neighborhood impact of the gardens [30,31,32]. This network analysis resulted in a range of 0–22 community gardens within the distance threshold for lower-income block groups (those with per capita income below that of Brooklyn as a whole). This information was calculated at the block group level for all community gardens in three different ways: independent of the year it was founded; gardens that were founded in 2005 or later; and those founded in 2010 and later (see Figure 3).

**Figure 4:**
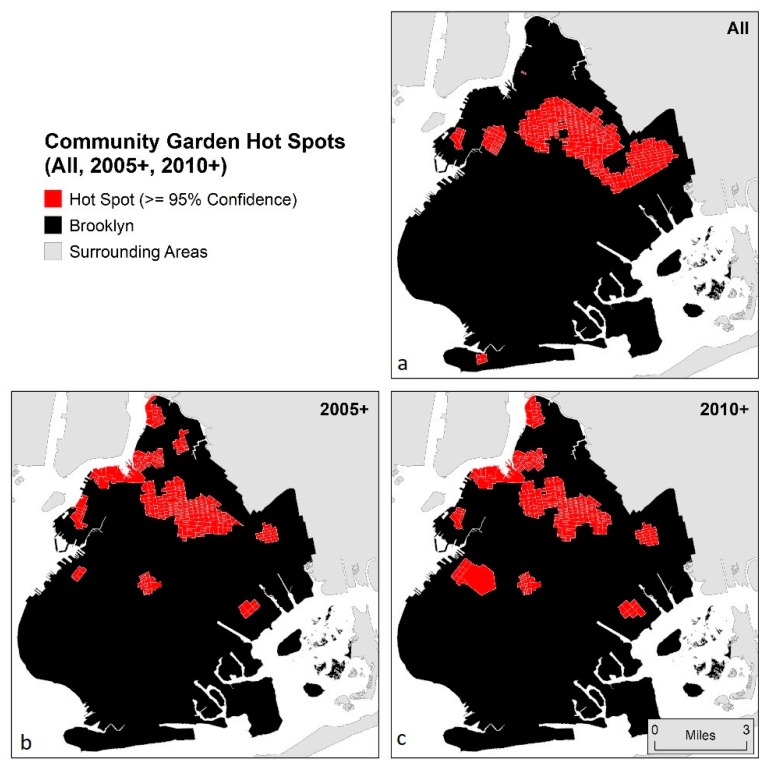
(**a**) Hot spots based on number of proximal community gardens founded any year;(**b**) hot spots based on counts of number of proximal community gardens founded in 2005 or later; and (**c**) hot spots based on counts of number of proximal community gardens founded in 2010 or later.

Hot spots (clusters of block groups with higher counts of proximal community gardens) were then calculated using the Getis–Ord Gi* statistic [64] with the distance threshold set at ¼ mile. This distance was selected to be consistent with the ¼ mile walkable neighborhood distance threshold of the network analysis, and to capture the spatial process of interest. Lower-income block groups with confidence values of 95% and above were retained for analysis. This output represents a characterization of proximity to community gardens at a different scale from the block group analysis, capturing a less localized version of the phenomenon (see Figure 4).

Differences in changes in per capita income between 2010 and 2015 for lower-income block groups with different proximity characteristics (e.g., in hot spots vs. not in hot spots; and with threshold distance proximity to community gardens vs. without proximity) were examined for all gardens, gardens founded in 2005 and later, and those founded in 2010 and later, using *t*-tests assuming unequal variances. This resulted in six different analyses of changes in per capita income.

Our analyses relied upon the use of the ACS dataset for income data and our derived change of income calculations between the two time periods of our study. The ACS is a sample representing a subset of the population, and is not a complete census. Several studies have pointed out the inherent error in the ACS estimates [65,66,67], and state that high margins of error (MOE) are more common with a smaller sample size and/or a small geographic area covered, leading to higher levels of uncertainty overall in the ACS data. The level of uncertainty varies widely from place to place. “An established and commonly used measure of uncertainty is the coefficient of variation (CV), which is the standard error of an estimate divided by the estimate itself.” [65] (p. 1537) By calculating the CV of each census unit, it can be seen where the data on a given variable is more reliable.

**Figure 5:**
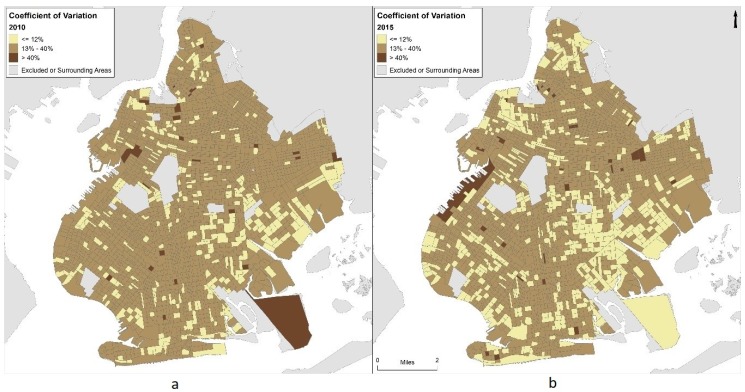
Coefficient of variation for American Community Survey five-year estimates of per capita income in 2010 (**a**) and 2015 (**b**).

To accommodate error inherent in the five-year ACS estimates, only block groups that had a coefficient of variance (CV) of high (CV ≤ 12%) or medium reliability (CV = 13–40%) for both 2010 and 2015 were included in the analysis (*n* = 1150) [67] (see Figure 5).

## 3. Results

**Figure 6:**
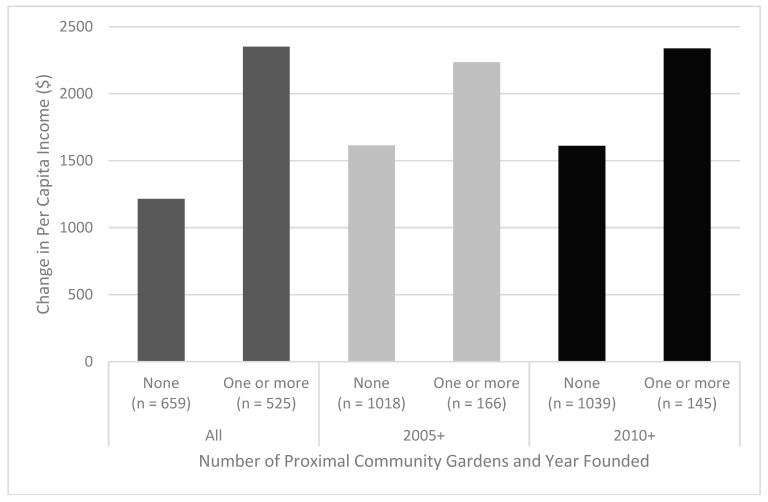
Population-weighted average change in per capita income (2010–2015) vs. proximity to one or more community gardens. Community gardens were categorized as founded in any year (all), those founded in 2005 or later (2005+), and those founded in 2010 or later (2010+).

Per capita income, Brooklyn-wide, increased from $25,493 to $26,774 in 2015-adjusted dollars, an increase of $1281. When examined at the block group level, there appears to be a larger increase in population-weighted per capita income between 2010 and 2015 when lower-income block groups are within the proximity threshold (≤¼ mile network distance) to at least one community garden when compared to lower-income block groups not near any community gardens (>¼ mile network distance) (see Table 1). This difference in population-weighted per capita income change attenuates when only newer community gardens are considered (those established since 2005 or 2010) (see Figure 6).

**Figure 7:**
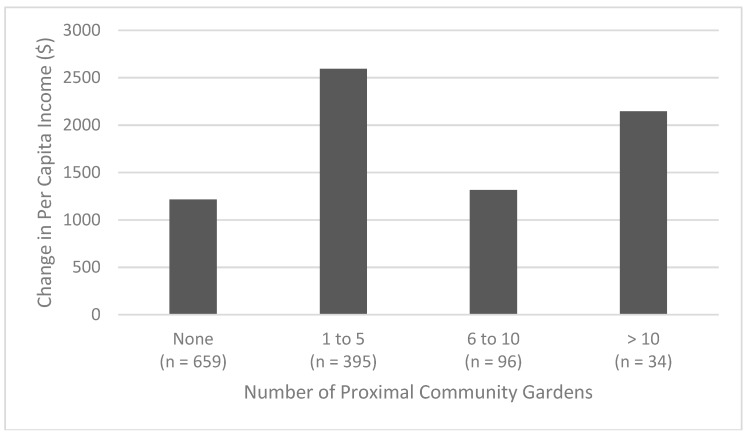
Population-weighted average change in per capita income (2010–2015) vs. number of community gardens proximal to lower-income block groups.

However, the relationship between change in population-weighted per capita income and number of proximal community gardens does not appear to be linear, based on a visualization of the data (see Figure 7). The relationship between income and nearby community gardens was not explored for the more recently founded gardens (those founded since 2005 or 2010). This was due to the small number of lower-income block groups with proximity to greater than one recently-founded community garden (only 3.4% of low-income block groups had more than one community garden founded since 2005, and only 1.9% for those founded since 2010) and the low maximum counts of proximal community gardens per block group (no single low-income block group had more than four proximal community gardens founded since 2005, and none had more than two founded since 2010).

Block group level *t*-tests suggest that proximity to one or more community garden is associated with significant increases in per capita income in lower-income census block groups between 2010 and 2015. However, that association is considerably weaker and loses significance when only including more recently founded gardens (2005+ and 2010+) (see Table 2).

At a more generalized (less localized) level, hot spot analyses (Gi*with confidence ≥ 95%) based on the number of community gardens founded in any year within ¼ mile network distance to census block group did not result in statistically significant *t*-tests with respect to the lower-income block group being located within hot spots and change in per capita income between 2010 and 2015. However, the magnitude of the associations increased, and significance was achieved, when only more recently established community gardens were considered (*p* < 0.1 and *p* < 0.05 for 2005+ and 2010+, respectively) (see Table 2). This phenomenon may be a function of scale, as the “all community garden” cluster is much larger than the clusters of more recently founded community gardens (237 lower-income block groups are inside the “all” cluster, and only 173 and 171 are in the 2005+ and 2010+ clusters, respectively) (see Figure 4).

## 4. Discussion

The research question posed in this study, “Is proximity to community gardens in less affluent neighborhoods associated with an increased likelihood of gentrification?”, was examined in two different ways: a census block group proximity analysis, and a hot spot analysis, to determine the potential impact of proximity to community gardens on lower-income block groups.

The results of the block group analysis suggest that proximity to community gardens is associated with increases in per capita income. This increase in per capita income over the study period (2010–2015) is indicative of areas which are either undergoing gentrification or which are at some later point in the gentrification process. The most noteworthy increases in per capita income were block groups that were proximal to at least one community garden. However, proximity to multiple community gardens (e.g., 5 or more and 10 or more) results in much less of an increase. When only newer gardens were examined (those founded in 2005 or later cumulative, or those founded in 2010 or later), the increase in per capita income was not significant. This may be because more recently founded community gardens have not been in existence long enough to attract gentrification to their neighborhoods, or it may reflect some difference in community activism and resistance to gentrification amongst the areas with more recent community gardens. Additional data and analyses are required to ferret out the reasons this is so.

A hot spot analysis was conducted in order to cluster the block groups based on the number of community gardens within ¼ mile. This analysis did not produce statistically significant results when all community gardens were included, suggesting that lower-income block groups within hot spots have similar increases in per capita income when compared to those outside the hot spots. However, the analysis did show significantly higher increases in per capita income in hot spots when only more recently founded gardens were considered. Although the results of the hot spot analysis examining community gardens founded “in any years” do not strongly support the results of the block group analysis, this apparent discrepancy may be explained by a number of factors, such as differences in scale of each analysis, variations in conceptualization of proximity between the two methods, and disparities in neighborhood social capital. Again, further study would help determine the correct explanations.

When the lower-income block groups are grouped together spatially, this may be protective against gentrification and as such not show a significant difference in increases in per capita income when compared to lower-income block groups outside of the hot spots. The way gentrification tends to expand is from the outer edges inward, or starting adjacent to higher-status areas and diffusing away. Therefore, these larger contiguous areas of lower-income in Brooklyn, even those having proximity to many community gardens, may not be as vulnerable to gentrification, because they are “protected” by the outer extent of lower-income areas. However, these are generalizations of how the hypothetical gentrification frontier may present itself, and it can vary significantly from locale to locale, depending, for instance, upon whether there is a specific revitalization amenity (e.g., a waterfront revitalization project) that may be jump-starting the gentrification process, which then emanates from that location, uni-or multi-directionally, based on the geographic features of the area. In other places, there is a “block-by-block micro-geography” in play, resulting in a more fragmented or discontinuous frontier. As well, highly segregated neighborhoods versus very ethnically diverse areas also influence the paths that gentrification takes [11,13,68,69]. The existence of many community gardens within lower-income neighborhoods may reflect (or produce) the relative higher social cohesion in these areas, and as such may be able to help a community resist gentrification. This may also help to explain the non-linear relationship between number of community gardens proximal to a block group and increases in per capita income found in the block group analysis.

An important limitation to this study is the error inherent in the ACS block group estimates for per capita income. Even though five-year estimates were used, there are still large margins of error (MOE) present. Additionally, these errors may be larger for lower income block groups [65]. To accommodate this, we limited the *t*-tests presented in this study to block groups with “medium” or “high” reliability based on coefficient of variability (CV ≤ 40%, *n* = 1150) [67]. *t*-tests were also performed using other inclusion criteria to test for robustness of the associations. When only block groups that had lower MOE terms for both 2010 and 2015 than the change in per capita income were included (*n* = 305) *t*-test outputs were similar with respect to directionality, magnitude, and significance (slightly stronger in the block group level analysis, and slightly weaker in the hot spot analysis). However, when more exclusive criteria were used (e.g., only block groups with CV values ≤ 12%) the directionality was not consistent, and results were not significant, likely due at least in part to the relatively small sample size (*n* = 48 rather than 1150). Future research may want to explore using larger geographic aggregates in order to reduce the impact this error [65,66,67].

This study adds a quantitative perspective on previous researchers’ more qualitative studies on the relationship of community gardens to gentrification. Community gardens may have an impact on gentrification, as demonstrated by our analyses, but the strength of the relationship found here, while showing some statistical significance, is quite modest. To be able to obtain more definitive results, future studies will need to take other factors into account, with a view towards developing a more comprehensive predictive model of gentrification itself, which might include such variables as historical settlement patterns and development trends, housing market conditions, transportation and other infrastructure, as well as other large “greening” initiatives by governmental or private developers.

Some questions that ought to be considered for future research are: Are community gardens part of the “just green enough” approach to hindering gentrification, or do community gardens in fact help instigate gentrification? Can we have environmental justice with regards to adequate community green space in less affluent areas without the adverse impacts of gentrification on these communities? If some amount of gentrification is unavoidable, how can we best cope with it to assure an acceptable environmental justice outcome?

Proponents of the “just green enough” approach offer some recommendations for preventing or at least minimizing the impacts of environmental gentrification. This approach should include making “room for continued industrial use and blue-collar work, where cleanup does not automatically or exclusively lead to the ‘parks, cafes, and a river walk’ model of a green city” [52] (p. 1028). There are ways for communities to actively resist environmental gentrification, or at least to avoid accelerating it. “Over the past few years, a new trend has emerged in direct response to the problem of eco-gentrification. I will label it ‘conscious anti-gentrification’. This kind of greening project aims to increase the environmental quality and public health of a neighbourhood but without changing its socio-economic character. This is done by explicitly rejecting elements that tend to lead to gentrification, such as fancy waterfronts; by including neighbourhood residents in the planning process; and by implementing changes gradually” [70] (p. 1).

Above all, greening efforts and urban sustainability initiatives need to incorporate social equity goals as a major component of any project [71]. Government needs to significantly contribute to the effort towards social equity by instituting and implementing policies that stabilize communities and prevent rapid gentrification, by means of affordability protections for residents and businesses; anti-gentrification rental controls; accommodations within zoning ordinances to prevent new development inappropriate to the existing context of the neighborhood and encourage conscious restorations and rehabilitating of existing older housing stock, and financial incentives for homeowners and landlords to do so, with built-in protections for existing residents; mixed use zoning and human-scaled buildings; smaller development projects at scattered sites rather than large mega-projects; new housing types geared toward existing populations of families (larger dwelling units, fewer studios and one bedrooms); limited equity “co-operative” housing; incorporating “nature” more seriously into all urban planning, in all parts of the city (NYC’s Million Trees initiative is a good example of this) and not just as an afterthought or as part of a profit-making scheme.

Green gentrification has implications for environmental justice because existing lower-income residents are likely to be displaced after their community is improved environmentally. “A sustainable development paradigm that addresses the social imperative of sustainable community development in the form of equity and livability should not be building sustainable neighbourhoods for only the higher-income subsection of the population either passively or actively through the displacement of lower-income families. Sustainable development, if it is actually to be sustainable, should not be for some, but for all” [48] (p. 679). The goal should be for the regeneration of neighborhoods through revitalization, rehabilitation, and/or replacement of aspects of the physical environment that are not working well, including housing stock and environmental amenities, but without the replacement of the people who live there.

## 5. Conclusions

There is a growing concern that neighborhood “greening” of lower-income areas results in gentrification, displacing existing residents and businesses, and replacing them with more affluent populations. Community gardens may be a contributing factor to changing socioeconomic characteristics of the neighborhood, for instance increases in income, which is considered to be one indicator of gentrification.

Using Brooklyn, New York as a case study area, spatial analyses were conducted to explore the relationship between proximity to community gardens and changes in per capita income over the time period 2010–2015. The block group analysis showed that proximity to at least one community garden is associated with significantly larger increases in per capita income during the study period. The hot spot analysis did not show the association as conclusively as the block group analysis, but still suggests a similar association between proximity to community gardens and increases in income.

This is among the first studies to spatially analyze and quantify the relationship between community gardens and increases in income as a metric of gentrification, with a view towards considering the role of community gardens as a “just green enough” strategy. Whether community gardens can be thought of in this way to mitigate the impacts of gentrification remains ambiguous based on the results of this study. On the one hand, there were significant income increases in areas in close proximity to community gardens, bolstering the conclusion that gentrification may be taking place, despite any potential counteracting effect of the community gardens as a “just green enough” solution. On the other hand, proximity to community gardens is only one of many possible factors that influence the onset of the gentrification process, and community gardens may in fact help to ameliorate, impede, or at least slow down the pernicious impacts of gentrification, whilst providing a valuable environmental benefit to the existing community.

In the worthy quest to transform contaminated or otherwise unused urban land into beneficial green space, we must acknowledge and never lose sight of the fact that these greening actions tend to pit the goals of environmental justice against the effects of environmental gentrification. Environmental justice seeks to ensure that everyone has equal access to environmental benefits and the means to achieve healthy lives, whilst also preventing some populations from bearing a disproportionate exposure to environmental burdens from noxious land uses. Environmental justice is especially aimed at protecting the poor, minority and immigrant communities, and other vulnerable populations, who currently disproportionately suffer the brunt of environmental burdens. Environmental gentrification, meaning gentrification as an outcome/product/consequence of the greening efforts and other environmental improvements in lower-income communities, often results in the benefits of such greening being shifted to the incoming affluent populations, and causing the poor and more vulnerable existing populations to be additionally burdened through displacement from the newly improved neighborhood into worse environments. Environmental gentrification, therefore, can be seen as contrary to and negating the mission and possible realization of environmental justice, in very real and demonstrable ways.

## Figures and Tables

**Table 1:** Population-weighted average change in per capita income (2010–2015) based on ¼ mile proximity to community gardens (CG) for all gardens (all), those founded in 2005 or later (2005+), and those founded in 2010 or later (2010+).

Founding Year	Proximal Community Gardens	Number of Block Groups	Change in Per Capita Income	Difference in per Capita Income Change
All	None	659	$1214	$1136
	One or more	525	$2350	
2005+	None	1018	$1613	$622
	One or more	166	$2236	
2010+	None	1039	$1611	$727
	One or more	145	$2338	

**Table 2:** *t*-tests of change in per capita income (2010–2015) for block group level and hot spot analyses. The block group level analysis represents lower-income census block groups with access to one or more community gardens vs. lower-income block groups with no gardens within ¼ mile. Hot spot analysis represents lower-income census block groups inside hot spots vs. those outside hot spots. Both analyses were performed multiple times based on the year the gardens were founded (all, 2005+, and 2010+) and excluded block groups with coefficients of variation larger than 40%.

Community Gardens Based on Founding Year	Block Group Level Analysis		Hot Spot Analysis	
	*t*-Statistic	*p*-Value	*t*-Statistic	*p*-Value
All Community Gardens	2.947	0.003	0.065	0.949
Community Gardens (2005+)	1.591	0.113	1.676	0.095
Community Gardens (2010+)	1.583	0.115	2.138	0.034
